# Steam Explosion Pretreatment of Lignocellulosic Biomass: A Mini-Review of Theorical and Experimental Approaches 

**DOI:** 10.3389/fchem.2021.705358

**Published:** 2021-11-11

**Authors:** Isabelle Ziegler-Devin, Laurent Chrusciel, Nicolas Brosse

**Affiliations:** Université de Lorraine, INRAE, LERMAB, F-54000, Nancy, France

**Keywords:** steam explosion, pretreatment, severity factor, explosive decompression, alternative calculation

## Abstract

Steam Explosion (SE) is one of the most efficient and environmentally friendly processes for the pretreatment of lignocellulosic biomass. It is an important tool for the development of the biorefinery concept to mitigate the recalcitrance of biomass. However, the two distinct steps of SE, steam cracking and explosive decompression, leading to the breakdown of the lignocellulosic matrix have generally been studied in empiric ways and clarification are needed. This mini-review provides new insights and recommendations regarding the properties of subcritical water, process modeling and the importance of the depressurization rate.

## Introduction

The 21st century is witnessing the emergence of a new industry based on lignocellulosic biomass refining. Lignocellulosic (LC) biomass is composed of intertwined carbohydrate polymers and lignin forming a complex and recalcitrant matrix (Shen and Sun, 2021). The goal of the pretreatment process is to open up the structure of lignocellulose and primarily to make the cellulose amenable to enzyme conversion. In addition, the recovery of non-cellulosic components (lignins, hemicelluloses) for an optimum valorization of biomass can also be one of the objectives. However, because of the naturally resistant carbohydrate-lignin shield, this step is energy consuming and required high capital costs. Many chemical or physico-chemical pretreatment technologies have been developed but a very limited number of processes has been reported as being potentially cost-effective for further industrial developments (Silveira et al., 2015; Yoshida et al., 2019; Soltanian et al., 2020).

Steam explosion (SE) is one of the most promising mechanico-physico-chemical pretreatments owning to its limited energy consumption and its low environmental impact (Jacquet et al., 2015). This process uses saturated steam at high pressure, injected into a batch or continuous reactor with lignocellulosic biomass for a short duration time (a few minutes). The SE pretreatment process can be divided into two independent steps 1) a steam boiling phase and 2) an explosion phase. The temperatures involved in this first stage are around 170°–210°C in order to provoke hydrolytic breakdown of the LC matrix. The second stage of the process corresponds to a conversion of thermal energy into mechanical energy. It involves a sudden pressure drop leading to a vapor expansion inside the fibres and a disruption of the fibrous structure. It has been reported that SE involved a strong alteration of LC including an increase of the surface area and porosity, a relocation of lignin and modification of its structure, a partial depolymerization and solubilization of hemicellulose and a strong increase of cellulose enzymatic digestibility (Auxenfans et al., 2017). As a result, this process is currently developed at pilot and/or industrial scales for different applications (ethanol and platform molecules by fermentation, energy densification and black pellets, fibers production.) (Rahardjo et al., 2021).

Although SE has been in use for some time and for various applications, the control of the process parameters is not properly mastered, detailed mechanisms are still poorly understood and process optimizations are carried out on an empirical basis. During the first step of the process, high pressures applied promote the diffusion of sub-critical water into the LC biomass and the hydrolytic breakdown of the cell wall components. The kinetics of hydrolysis of these LC components are correlated to the residence time of the biomass in the reactor and to the temperature applied. The two parameters time and temperature being correlated, severity factors which combined them have been proposed in the literature. These factors are currently broadly employed in order to facilitate the control and the optimization of the SE process (Overend and Chornet, 1987; Fockink et al., 2018; Bonfiglio et al., 2019; Obame et al., 2019). However, these factors are based on semi-empirical kinetic approaches and assumptions that require discussion and clarification. The second stage consists in a sudden depressurization leading to a rapid re-volatilization of water contained in the LC material. The pressure difference between the pressurized reactor and atmospheric pressure in the explosion tank is then an important feature. Most of the publications dealing with SE assume that the shear forces applied to the biomass are solely a function of the pressure applied during the cooking stage. However, the depressurization time also appears to be an important parameter that can strongly impact the physical effect of the explosion. The depressurization is a complex phenomenon, linked to the technical characteristics of the equipment and is rarely mentioned. The objective of this mini-review is then to clarify some theoretical and experimental concepts of Steam Explosion pretreatment in order to improve the efficiency of this process.

## Water State and Properties in a Steam Explosion

During the hydrothermal treatment of lignocellulosics, three water states can be distinguished: the bound water in the cell wall which acts as a swelling agent, a plasticizer and the free water also called bulk water and free steam water. Generally, SE pretreatment is performed after a presoaking step and the actual amount of water injected into the form of steam into the SE reactor to reach the set temperature and pressure is not well controlled. Sui and Chen (Chen and Sui, 2015; Chen and Sui, 2016) examined by TD-NMR the role of water states in SE of corn stalk. They concluded that the optimum water content for SE corresponded to the fiber saturated point for which the cell wall is saturated but the cell cavities are empty, the free water displaying a buffering effect and leading to a higher energy consumption.

**FIGURE 1:**
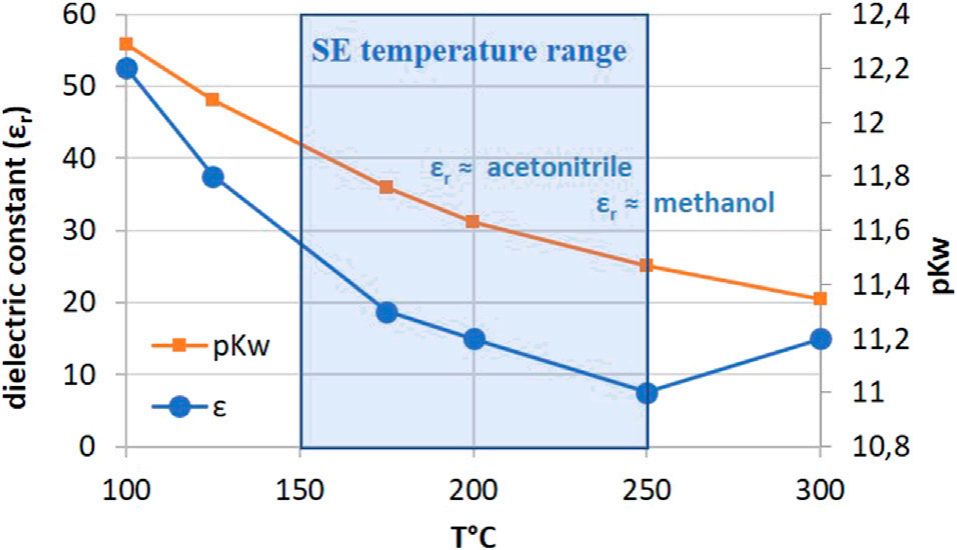
Evolution of the relative dielectric constant of water with the temperature.

At the conditions of SE, the pressurized water is called super-heated or subcritical water. Subcritical water is liquid water under pressure at a temperature between 100°C and 374°C with particular properties including lower dielectric constant. Water at room temperature is a polar solvent with a dielectric constant (ε_r_) of 79.9. At 170°C, the electric constant of water is close to that of acetonitrile (ε_r_ = 36) and at 220°C closed to that of methanol (ε_r_ = 27) (Figure 1). The other characteristics of subcritical water are lower diffusivity and viscosity and higher self-ionization and solvating power. As a result, the high temperature/low duration conditions of SE allow the extraction of thermally labile mid-polar compounds such as phenolics or polysaccharides with a low degradation rate due to the short reaction times. Recent examples from literature include extraction of sterols and phenols from olive pomace (Ustundag et al., 2018), bioactive compounds from strawberry extrudate (ascorbic acid, anthocyanins, ellagitannins, different vitamins, carotenoids, folic acid, and flavonoids) (Munoz-Arjona et al., 2020), highly acetylated mannanes and xylanes from wood (Mougnala Moukagni et al., 2021; Chadni et al., 2019).

The higher ionization product of subcritical water (K*w* < 10^−14^) also provides a more acidic environment. The evolution of pK*w* with temperature is given in Figure 1. At a temperature around 200°C, pK*w* ≈ 11 facilitates auto-hydrolysis reactions of biomass leading to a partial deacetylation and depolymerization of hemicelluloses, the cleavage of lignin inter-units and lignocellulosic complex and a reduction of cellulose DP. The combination of all these hydrolytic reactions leads to a significant degradation of the cell wall components and produces a cellulose-rich residue bearing a higher enzyme accessibility. Steam explosion can be also employed as an efficient and cost-effective pretreatment prior to a traditional solid-liquid extraction using water or alcohol. This approach has been used to recover bioactive compounds such as flavonoids, and phenolics (Kurosumi et al., 2007; Gong et al., 2012). Relatively harsh SE conditions are then required to destabilize the lignocellulosic complex to increase the extractability of the compounds (T = 190–260°C). In fact, the high extractability of molecules and polymers by SE is not only due to water state but also to the physical changes in the cell wall (i.e., increase of porosity) induced by the treatment, especially during the decompression step. This point will be discussed in *Explosive decompression*. However, these conditions may affect sensible bioactive molecules such as glycosylated flavonoids (Chen and Chen, 2011). In order to optimize the extraction of thermosensitive molecules, Fu and Chen described a low temperature steam explosion pretreatment (T = 80–160°C) with a partial pressure of steam (Fu and Chen, 2015). This air-steam explosion process was optimized for the extraction of chlorogenic acid from leaves.

## Steam Step: H-Factor, P-Factor and Severity-Factor

The severity factor is a parameter that aims to reflect the severity of the pre-treatment of a biomass by considering both the temperature of the pre-treatment and its duration through the residence time. The concept of the severity factor was indirectly determined by Vroom in 1957 through the “H factor” (Vroom, 1957). The H-factor is a variable used in the Kraft cooking process to combine temperature and time variables into a single variable representing the severity of the process. The Vroom’s H-factor (Eq. 1) is based on the Arrhenius’ law and assumes that 1) delignification takes place in a single reaction, 2) by arbitrarily setting a relative reaction rate constant (k_r_) equals to one at 100°C (373.15 K) and 3) that the constant activation energy for softwood is of the order of 134 kJ mol^−1^ based on Larocque and Maas’studies (Larocque and Maass, 1941).

$$H\left(t\right)=\overset{t}{\underset{0}{\int }}k_{r}dt\;=\overset{t}{\underset{0}{\int }}\frac{k_{T}}{k_{373}}dt=\overset{t}{\underset{0}{\int }}e^{\frac{134\;000}{8.314x373}-\frac{134\;000}{8.314\;x\;T}}\;dt\;\approx \;\overset{t}{\underset{0}{\int }}e^{43.181-\frac{16113}{\;T}}dt$$ (1)

Equation 1: Vroom’s H factor

In 1965 Brasch and Free introduced an equivalent concept (P-factor) for the optimization of the prehydrolysis step of *Pinus radiata* chips in the Kraft process for the production of pulp (Brasch and Free, 1965). The prehydrolysis step allows to obtain a higher cellulose content, to eliminate a maximum of hemicelluloses and to facilitate the subsequent bleaching of the pulp. Since prehydrolysis or pre-extraction reactions involve the solubilization of hemicellulose oligomers rather than delignification as in the Kraft pulping process, the activation energies are slightly different leading to difference between H-factor and P-factor. Moreover, one should not forget that the H-factor is defined from a ratio of the rate constants (it does not consider the order of the reaction), while the P-factor is calculated from the ratio of the rates (it considers the order of the reaction). Autohydrolysis experiments of Brasch and Free were carried out in a pressurized batch reactor with reaction temperatures up to 170°C and hydrolysis times up to 2 h. The authors observed that in the case of the autohydrolysis of Radius Pine, the autohydrolysis rate is approximatively multiplied by three when the hydrolysis temperature increases by 10°C. Choosing a relative reaction rate of unit reference at 100°C, they mathematically translated this tripling of rate, which is similar to a temperature coefficient, by the following geometrical progression (Eq. 2)

$$\frac{relative\;rate\;\left(T_{r}\right)}{relative\;rate\;\left(100{}^{\circ}C\right)}=\;3^{\frac{T_{r}-100}{10}}$$ (2)

Where Tr is the hydrolysis temperature in °C, 100°C is the reference temperature, relative rate at 100°C = 1 (without unit)

Equation 2: Calculation of the relative hydrolysis rate of Radius Pine according to Brasch and Free.

P-factor, defined by Brasch and Free (1965), is related to the area under the curve of the relative rate vs residence time. P-factor calculation is based on a temperature gradient of 20°C–170°C during the first 40 min of hydrolysis and an isothermal temperature of 170°C for 60 min.

P-factor can therefore be calculated according to Eq. 3. It was then shown that the H-factor shows a very good correlation with the prehydrolysis degree obtained as well as with other characteristics (lignin content, pH, Kappa index, intrinsic viscosity...) of the final Kraft pulp for different experimental conditions.

$$H\;=\;\overset{t}{\underset{0}{\int }}3^{\frac{T_{r}-100}{10}}\;dt$$ (3)

Equation 3: Calculation of H-factor in the case of Radius Pine.

The P-factor developed by Brasch and Free (1965) does not make any assumptions about the hydrolysis mechanism, nor about the reaction(s) order. It is only based on an experimental observation of the temperature coefficient. It should be noted that the range of variation of *P* value is strongly influenced by the unit of time chosen. This factor therefore combines three parameters: a temperature coefficient (here of 3) which has been determined by the experiment, the reaction temperature, the residence time.

Based on the work of Brasch and Free, Overend and Chornet (1987) proposed the calculation of the severity of pre-treatment in the case of steam explosion using Eq. 4. This formula is a simple mathematical variation of the P-factor, which assumes that the temperature coefficient (TC) is two and that the temperature does not vary with the residence time. Contrary to the publication of Brasch and Free, Overend and Chornet did not justify the assumption on the temperature coefficient.

$$S{}^{\circ}\;=\;t_{s}\;x\;e^{\frac{T_{r}-100}{14,75}}\;\;$$ (4)

Where t_s_ is the residence time, usually expressed in minutes, Tr the reaction temperature in °C, 100°C the reference temperature.

Equation 4: Calculation of the severity factor of a steam explosion pretreatment according to Overend and Chornet.

Eq. 4 is currently broadly used in the literature, in logarithmic form (Log S°), to determine the severity of pretreatments. This equation which includes temperature and residence time is primarily giving a valuable estimation of the impact of the pretreatment on the hemicellulose hydrolysis and enzymatic saccharification. This model is no longer applicable when a catalyst (acidic or basic catalyst) is used, the pH of the medium strongly affecting the lignocellulosic breakdown kinetics and mechanisms. As a result, an adaptation (Combined Severity Factor) including the pH is also widely used (Eq. 5) (Chum et al., 1990).

CSF=logS°-pH (5)

Equation 5: Calculation of a Combined severity Factor.

Other applications than hemicelluloses hydrolysis have also been reported including SE lignin extraction (Zhang et al., 2019; Obame et al., 2019), carboxylic acids and pellets production (Sharma and Dubey, 2020). This model has also been used to predict the reactivity of biomass using different pretreatment technologies such as organosolv (Goh et al., 2011), dilute acid (Ilanidis et al., 2021) or alkaline treatment (Wang et al., 2021).

However, several works have shown that samples treated at the same CSF or SF displayed differences in chemical composition, in hydrolysablility or in morphological structure. Fockink et al. (2018) reported that sugar cane bagasse steam exploded at equivalent CSF values at T > 195°C in absence or in presence of acid produced lignocellulosic pulps with different composition and accessibility to enzymes. Similar observations have been made by Esperito Santo et al. (2020) and these authors suggested that CSF modeling could be not robust enough for a reliable prediction of acid-catalyzed steam explosion. Moreover, for alkali treatment the relevance of this approach can be questioned given the fact that the deconstruction mechanisms of biomass are very different at high pH (peeling reaction, basic hydrolysis and saponification reactions … ).

In Eqs 4, 5, many approximations have been made on the interpretation of the physical meaning of factor 14.75 used in the Overend and Chornet’s severity factor calculation. Contrary to what is often claimed this factor does not correspond to an activation energy but mathematically reflects the temperature coefficient of two. It should be noted that this formula is almost equivalent to the expression (Eq. 6) from the progression proposed by Brasch and Free (1965) using the same temperature coefficient:

$$S{}^{\circ}\;=\;t_{s}\;x\;2^{\frac{T_{r}-100}{10}}$$ (6)

Where ts is the residence time, usually expressed in minutes, Tr is the reaction temperature in °C. 100°C is the reference temperature.

Equation 6: Alternative calculation of the severity factor of a steam-explosion pre-treatment - adapted from Brasch and Free (1965).

Eq. 6 appears to be more appropriate than Eq. 4 as it clearly implies an estimated value of two for TC, which has a real physical meaning. In order to consider, the fact that the TC varies strongly according to the parameters of the experiment (nature of the biomass, granulometry, pre-treatment and impregnation conditions), an alternative model is proposed below.

## Explosive Decompression

There are two processes for steam explosion: the batch process and the continuous process. In batch steam explosion facilities, the hydrothermally treated biomass is subjected to a brutal depressurization by the sudden opening of a valve causing the material to be transferred from the reactor to the discharge tank. For continuous steam explosion systems, the biomass is continuously fed into the pressurized reactor and conveyed through the digester by an auger but the explosion remains a discontinuous phenomenon. An opening valve, regulated by detecting the torque on the last transfer screw or by a timer causes repeated small explosions. Thus, whatever the system used, continuous or batch, the explosive depressurization step has very similar characteristics (Lam, 2011). According tu Yu et al., the explosion step of the steam explosion process, approximates an adiabatic expansion process and induces a conversion process of thermal energy into mechanical energy (Yu et al., 2012). Thus, the explosive decompression of SE induces shear forces and creates microcracks in the cellular structure and a fragmentation/defibration of the lignocellulosic material (Sun et al., 2005; Muzamal et al., 2016). Holtzapple et al. (1989) studied the energy requirement for size reduction of wood using steam explosion compared to conventional milling methods. The results showed that the steam explosion process requires 70% less energy to achieve the same size reduction as the mechanical method (Holtzapple et al., 1989). The authors showed that by steam explosion an increase in surface area of 12.9 m^2^ kg^−1^ of aspen and poplar wood required an equivalent heat of 1.63 MJ kg^−1^.

Processes described in the literature referring to steam explosion generally use temperatures of 160°C–260°C of saturated steam which correspond to a pressure range of 5–45 bar (Chen and Liu, 2015). The impact of this explosive expansion on the biomass and the mechanical effect induced are not considered in the severity factor modeling previously described (see *Steam Step: H-Factor, P-Factor and Severity-Facto*r). For example, two SE carried out one at 160°C for 35 min and the other at 200°C for 2 min have the same severity factor (Log S° = 3.3) whereas the pressure drops will be far different (ΔP = 5.2 and 14.5 bars respectively). The induced shear forces will therefore be much higher in the second case.

The specific contribution of the decompressive expansion has been examined by several authors. Pielhop el al. studied the influence of this step on the enzymatic digestibility of different biomasses. They showed that explosive decompression improved digestibility mainly due to particle size reduction (Seidel et al., 2017). Due to this size reduction effect, lower amounts of enzyme are required and especially recalcitrant species like softwood became digestible by enzymes (Pielhop et al., 2016). However, the quick decompression does not seem to have a significant effect on the structure of the polymers, it does not promote the depolymerization of hemicellulose or lignin (Rodríguez et al., 2017). The physical changes due to water expansion and the increase in cell wall porosity have been investigated using microscopic techniques and fluorescent probes (Kvist et al., 2018) or physico-chemical approaches (Sui and Chen, 2014). It has been shown that SE greatly affected the porosity and diffusion of molecules (Qin and Chen, 2015) and macromolecules (Kvist et al., 2018) by increasing the size, the connectivity and tortuosity of the pores. Wood fiber deformation was simulated using finite element modeling. It was shown that the parietal impact is greater on the outer layers (P and S1) of the wood cell and that thin-walled cells (earlywood) and cells with low microfibrillar angle were the most impacted (Muzamal et al., 2014). Experimental and numerical modeling work has shown structural changes in wood cells during steam expansion, including cell deformation and microcracks formation (Muzamal et al., 2017; Zhang and Cai, 2006; Feng et al., 2016). The effect of these morphological modifications on some physical properties (i.e. permeability, sound absorption capability) of the material was examined (Kang et al., 2010).

Physically the concept of explosion is defined as a rapid transition of the state of matter accompanied by a sound phenomenon of explosion, linked to the sudden release of energy. During pre-treatment by steam explosion, the deflation time must be shorter than the pressure equilibrium time between the inside and outside of the internal structures of the pre-treated biomass (Yu et al., 2012). According to Yu et al., the duration of the explosion can be explained by two interdependent time periods: 1) the opening time of the ball valve and 2) the equilibration time of the steam pressure contained in the reactor to the atmosphere, which is correlated to both the volume of steam and the cross-sectional area of the valve. The total “deflation” time, proposed by Yu et al., is calculated from the characteristic equations of fluid mechanics and corresponds to the sum of three terms: the valve opening time, the sonic deflation time and finally the subsonic deflation time : (expressed in seconds).

$${\text{t}}_{\text{tvalve opening}}:\text{valve opening time (data provided by the manufacturers)}.{\text{t}}_{\text{sonic deflation }}=\frac{2\text{k}}{\text{k}-1}\left[{\left(\frac{{\text{P}}_{1}}{{\text{P}}^{\text{*}}}\right)}^{\frac{\text{k}-1}{2\text{k}}}-1\right]\text{x }5,217\;\frac{\text{V}}{\text{kS}}\sqrt{\frac{273}{{\text{T}}_{\text{s}}}}$$

$${\text{t}}_{\text{subsonic deflation }}=0,945\;{\left(\frac{{\text{P}}_{1}}{0,1013}\right)}^{\frac{\text{k}-1}{2\text{k}}}\text{x }5,217\;\frac{\text{V}}{\text{kS}}\sqrt{\frac{273}{{\text{T}}_{\text{s}}}}\;$$ (7)

With, P_1_: absolute pressure of the process in MPa, P*: critical pressure with a common value for 0.192 MPa (at the sonic point), k: Laplace coefficient; k = 1,33 for steam at 20°C, V: Steam volume in the reactor in L, S: Ball valve cross-sectional area in mm^2^, T_s_: Vapor temperature in K.

Equation 7: Calculation of the explosion duration according to Yu et al. (2012).

Thus, if the decompression is fast enough, most of the steam and hot liquid water contained in the biomass expands rapidly and releases from the lignocellulosic structure, inducing mechanical shear forces. There is no threshold value proposed in the literature for the decompression step but if it is too slow, the pressure has time to equalize across the structure leading to a limited or even non-existent decompression effect.

At the scale of the process, this phenomenon of explosive decompression is correlated to the nature but also to the size of the blow-off valve. Most of the existing batch pilots use ball valves, which have the advantage of freeing the entire section of pipe at the reactor outlet in the open position while ensuring a very low pressure drop and thus a high Kvs value. Valve sizing and automation is also one of the limitations of scaling the SE process, as the larger the ball valve cross section, the slower the opening speed. A major difficulty in the study of the decompression step is that the parameters of the fast opening valves (diameter and especially opening time) are generally not mentioned in the articles; however, they can be provided by ball valves manufacturers. For example, the opening speed of a ball valve is ≈0.8 s for a 2-inches diameter and more than ≈2 s for a 12 inches diameter. The total duration of the explosion is also strongly dependent on the volume of the reactor and the cross-section of the valve. For a laboratory scale 4 L pilot reactor equipped with a 2-inches ball valve and operating at 220°C (2.3 MPa), the total “deflation” time is estimated to be 0.83 s, with the ball valve opening time being much longer (0.8 s) than the decompression time (0.03 s). On the other hand, at the same temperature/pressure conditions, for a 50 m^3^ semi-industrial scale reactor equipped with a 10-inches ball valve, the total “deflation” time is ≈15 s. This high opening time tends towards slow expansion conditions limiting the interest of the SE process (Yu et al., 2012).

## Conclusion and Recommendations

SE, which is a combination of a sub-critical water treatment and a cell wall disruption, has been extensively described as a pretreatment for the production of fermentable sugars. The specific characteristics of the SE process (including water low dielectric constant, short residence time, chemical-free extraction) make it a method of choice for the isolation of high-value compounds such as phenolics or polysaccharides from biomass. These types of applications deserve to be further developed as green alternative to conventional solvent extraction methods. However, for heat-sensitive and hydrolysable compounds, the high temperature conditions commonly used in SE require detailed optimization.

Biomass is characterized by an extremley high variability requiring optimization of the treatment used for its fractionation. Single severity factors which combined in one equation the most SE influential parameters (Eqs 4, 5) are broadly utilized in literature. However, even if these equations could be useful in many cases to compare and optimize treatments, they are based on semi-empirical kinetic approaches. For a more appropriate model we recommend to determine for each experiment the temperature coefficient (TC) which is function of the nature of the biomass, its granulometry and the pH of impregnation. For this, as a first approximation, the ratio of the average mass loss rates for each 10°C increase between different experiments should be calculated. The severity factor would then be calculated according to:

$$S{}^{\circ}\;=\;t_{s}\;x\;{\text{TC}}^{\frac{T_{r}-100}{10}}$$ (8)

Where TC is the experimental temperature coefficient.

Equation 8: New alternative calculation of the severity factor of a steam-explosion pre-treatment.

$$TC\;=\;\frac{{\bar{\text{r}}}_{\left(\text{T}+10\right)}}{{\bar{\text{r}}}_{\left(\text{T}\right)}}$$ (9)

Where ${\bar{r}}_{\;}\;$ is the average mass loss rate.

Equation 9: TC calculation.

As far as explosive decompression is concerned, the total duration of the explosion is generally not considered in the literature. However, it is an essential characteristic related to technical parameters. It is then recommended to check if the experimental pilot used meets the criteria of real explosion. Thus, a better control of SE parameters should allow an optimal use of this powerful process for a wide range of applications.
